# Changes in Payer Mix Associated With Private Equity Acquisition of Ophthalmology Practices

**DOI:** 10.1001/jamanetworkopen.2025.12629

**Published:** 2025-05-28

**Authors:** John E. Connolly, Matthew Guido, Anthony Girard, Robert Tyler Braun, Ezekiel J. Emanuel

**Affiliations:** 1 Department of Medicine, Johns Hopkins Hospital, Baltimore, Maryland; 2 Department of Medical Ethics and Health Policy, University of Pennsylvania, Philadelphia; 3 Department of Population Health Sciences, Weill Cornell Medicine, New York, New York

## Abstract

This cross-sectional study examines the changes in payer mix associated with private equity acquisition of opthamology practices in the US.

## Introduction

Private equity (PE) ownership of medical practices is associated with higher prices, greater use, and mixed evidence on quality.^1,2^ Less is known about PE’s association with payer mix, an important consideration as higher commercial payer mix enhances financial health but could minimize care of lower-income patients.^3^ We hypothesized that PE acquisition is associated with increased proportion and number of commercial claims per ophthalmologist.

## Methods

In this cross-sectional study we used a database of ophthalmology practices acquired by PE and identified ophthalmologists as PE-affiliated or non–PE-affiliated for each quarter between 2012 and 2022.^4^ Our analysis focused on ophthalmologists because they have attracted significant PE investment.^5^ Data were analyzed from January to December 2024. This study was exempt from oversight by the University of Pennsylvania institutional review board per the Common Rule and followed the Strengthening the Reporting of Observational Studies in Epidemiology (STROBE) reporting guideline. Consent was not required because of the retrospective nature of the study.

We used a unique multipayer claims database comprising over 40 million distinct commercial, Medicare fee-for-service (FFS), Medicare Advantage (MA), and Medicaid claims from 2018 to 2022, and categorized claims by rendering National Provider Identifier. Ophthalmologists affiliated with PE prior to 2018 were excluded. We stratified claims into new (CPT codes 99201 to 99205) and established (CPT codes 99211 to 99215) visits.

A difference-in-difference analysis was used to determine whether the number and proportion of claims billed by payer changed for PE-affiliated compared with non–PE-affiliated ophthalmologists following acquisition. Estimates were generated with a Callaway and Sant’Anna difference-in-difference estimator to address negative weights. To achieve improved balance in our sample, ophthalmologists must have been in a practice for at least 4 quarters before and after acquisition; thus our acquisition window was 2019 to 2021. The control group included ophthalmologists not acquired by PE (including after the study period) with at least 4 quarters worth of data. Standard errors were clustered at the practice level and the unit of analysis was claims per ophthalmologist per quarter. Fixed effects were included for Hospital Referral Region. Significance was defined as *P* < .05. Event study plots of dynamic effect estimates were created to test the parallel trends assumption (eMethods in Supplement 1).

## Results

A total of 504 ophthalmologists were PE-affiliated, and 11 085 were nonaffiliated. Following acquisition, PE-affiliated ophthalmologists had greater increases (Table 1) in established commercial claims (DID, 16.59 [95% CI, 5.91-27.27]), established Medicare FFS claims (DID, 12.35; [95% CI, 3.71-20.99]), and established Medicaid claims (DID, 2.66; [95% CI, 0.03-5.28]) relative to non–PE-affiliated ophthalmologists. PE affiliation was associated with an increase in proportion of established claims that were Medicare FFS and a decrease in proportion of established claims that were commercial (Table 2).

**Table 1.** Change in Claims Per Quarter Following Acquisition for Private Equity (PE)- vs Non–PE-Affiliated Ophthalmologists

Claim	Claims, No.				DID estimate (SE) [95% CI]	*P* value^a^
	Pre 2018		Post 2022			
	PE	Non-PE	PE	Non-PE		
New commercial claims per ophthalmologist	40.24	25.76	39.34	29.24	2.13 (1.99) [−1.78 to 6.03]	.29
Established commercial claims per ophthalmologist	83.49	57.70	136.45	86.75	16.59 (5.45) [5.91 to 27.27]	.002
New MA claims per ophthalmologist	2.46	0.58	2.26	0.81	−0.05 (0.23) [−0.50 to 0.40]	.83
Established MA claims per ophthalmologist	9.92	2.42	2.89	3.90	−1.28 (0.79) [−2.84 to 0.27]	.11
New FFS claims per Ophthalmologist	19.42	7.59	10.42	5.83	−0.76 (1.01) [−2.73 to 1.22]	.45
Established FFS claims per ophthalmologist	42.22	27.28	58.40	29.76	12.35 (4.41) [3.71 to 20.99]	.005
New Medicaid claims per ophthalmologist	1.62	1.78	2.21	1.75	1.05 (0.39) [0.30 to 1.81]	.006
Established Medicaid claims per ophthalmologist	3.62	3.74	4.51	4.35	2.66 (1.34) [0.03 to 5.28]	.047

Abbreviations: DID, difference-in-difference; FFS, Medicare Fee-For-Service; MA, Medicare Advantage.

^a^ Significant result with *P* < .05.

**Table 2.** Change in Proportion Claims Per Quarter Following Acquisition for Private Equity (PE)- vs Non-PE-Affiliated Ophthalmologists

Proportion claim	DID Estimate (SE) [95% CI]	*P* value^a^
New commercial	2.22 (1.12) [0.03 to 4.41]	.046
Established commercial	−3.43 (1.21) [−5.80 to −1.05]	.005
New FFS	−1.20 (0.85) [−2.87 to 0.46]	.16
Established FFS	3.29 (1.14) [1.06 to 5.53]	.004
New MA	−0.43 (0.43) [−1.28 to 0.42]	.32
Established MA	0.43 (0.51) [−0.56 to 1.43]	.39
New Medicaid	−0.59 (0.58) [−1.73 to 0.54]	.31
Established Medicaid	−0.30 (0.55) [−1.38 to 0.79]	.59

Abbreviations: DID, difference-in-difference; FFS, Medicare Fee-For-Service; MA, Medicare Advantage.

^a^ Significant result with *P* < .05.

In preacquisition time points, 95% CIs consistently included null effects across outcomes. Among outcomes found to have significant results, 95% CIs showed significant deviation from the null over time.

## Discussion

Following acquisition by PE, ophthalmologists experienced an increase in the number of established commercial, Medicare FFS, and Medicaid claims. PE acquisition was also associated with an increase in proportion of established Medicare FFS claims and decrease in proportion of established commercial claims.

The increase in established claims across payers was consistent with a strategy designed to increase organizational revenue. That proportion of established claims was more heavily weighted toward Medicare FFS and away from commercial claims was surprising given lower rates associated with Medicare. This may reflect commercial insurers shifting care away from PE-affiliated ophthalmologists or could suggest a desire by PE-affiliated ophthalmologists to retain Medicare FFS patients, potentially because of stable reimbursement rates. These results comport with evidence that ambulatory surgery centers shifted toward Medicare patients following PE acquisition.^6^ We did not analyze the change in distribution of CPT codes following PE acquisition; changes in coding practice could be an important focus of future studies.

## Notes

Supplement 1.
eMethods.

Supplement 2.
Data Sharing Statement
