# Supplementary material for: Changes in Payer Mix Associated With Private Equity Acquisition of Ophthalmology Practices
Source: JAMA Netw Open. 2025 May 28;8(5):e2512629. doi: 10.1001/jamanetworkopen.2025.12629 (PMC12120645; doi:10.1001/jamanetworkopen.2025.12629)
Supplement: Supplement 1. — eMethods. [file jamanetwopen-e2512629-s001.pdf]

Supplemental Online Content

Connolly JE, Guido M, Girard A, Braun RT, Emanuel EJ. Changes in payor mix associated with private equity acquisition of ophthalmology practices. *JAMA Netw Open*. 2025;8(5):e2512629. doi:10.1001/jamanetworkopen.2025.12629

eMethods.

This supplemental material has been provided by the authors to give readers additional information about their work.

© 2025 Connolly JE et al. *JAMA Network Open*.

eMethods.

The reference period for event study regressions was the preceding period in pre-acquisition periods and the period immediately before acquisition in the post-acquisition periods. We omit a reference period because the reference period is varying among treatment groups. This is the default option used in the difference-in-difference package by Callaway and Sant'Anna used by our study.

© 2025 Connolly JE et al. *JAMA Network Open*.
