# Supplementary material for: Changes in Payer Mix Associated With Private Equity Acquisition of Ophthalmology Practices
Source: JAMA Netw Open. 2025 May 28;8(5):e2512629. doi: 10.1001/jamanetworkopen.2025.12629 (PMC12120645; doi:10.1001/jamanetworkopen.2025.12629)
Supplement: Supplement 2. — Data Sharing Statement [file jamanetwopen-e2512629-s002.pdf]

Data Sharing Statement

Connolly. Changes in Payer Mix Associated With Private Equity Acquisition of Ophthalmology Practices. *JAMA Netw Open*. Published May 28, 2025. doi:10.1001/jamanetworkopen.2025.12629

Data
Data available: No
